# Cost-Effectiveness of Administering Rituximab for Steroid-Dependent Nephrotic Syndrome and Frequently Relapsing Nephrotic Syndrome: A Preliminary Study in Japan

**DOI:** 10.1038/srep46036

**Published:** 2017-04-07

**Authors:** Tomoyuki Takura, Takashi Takei, Kosaku Nitta

**Affiliations:** 1 Department of Healthcare Economics and Health Policy, Graduate School of Medicine, The University of Tokyo, Tokyo, Japan; 2 Osaka University Graduate School of Medicine, Osaka, Japan; 3 Department of Nephrology, Tokyo Metropolitan Geriatric Hospital and Institute of Gerontology, Tokyo, Japan; 4 Department of Medicine, Kidney Center, Tokyo Women’s Medical University, Tokyo, Japan.

## Abstract

With regard to the use of rituximab for patients with steroid-dependent nephrotic syndrome and frequently relapsing nephrotic syndrome, not only has the regimen not been clinically verified but also there is a lack of health economics evidence. Therefore, we conducted a prospective clinical study on 30 patients before (with steroids and immunosuppressants) and after introducing rituximab therapy. Relapse rates and total invoiced medical expenses were selected as the primary endpoints for treatment effectiveness and treatment costs, respectively. As secondary endpoints, cost-effectiveness was compared before and after administering rituximab in relation to previous pharmacotherapy. The observation period was 24 months before and after the initiation of rituximab. We showed that there was a statistically significant improvement in the relapse rate from a mean of 4.30 events before administration to a mean of 0.27 events after administration and that there was a significantly better prognosis in the cumulative avoidance of relapse rate by Kaplan–Meier analysis (*p* < 0.01). Finally, the total medical costs decreased from 2,923 USD to 1,280 USD per month, and the pre–post cost-effectiveness was confirmed as dominant. We, therefore, conclude that treatment with rituximab was possibly superior to previous pharmacological treatments from a health economics perspective.

Nephrotic syndrome is the generic name given to pathological conditions associated with proteinuria (≥3.5 g/day), hypoproteinemia, and generalized edema. The disorder is further classified into primary nephrotic syndrome caused by primary glomerular disease and secondary nephrotic syndrome caused by systemic disorders. When it appears during childhood, it is mainly the primary type and typically consists of minimal-change disease. By contrast, nephrotic syndrome in adults is distinguished by a wide variety of types, including membranous nephropathy, membranoproliferative glomerulonephritis, and other secondary types that may lead to lupus nephritis or diabetic kidney disease^1^. The syndrome rapidly improves with steroid (e.g., prednisolone) and immunosuppressant (e.g., cyclosporine) treatment. Refractory cases may also occur (frequent relapse type, steroid dependence, or steroid resistance) that require steroid therapy for prolonged periods of time and for which side effects become a major issue. There are, therefore, calls for novel medical strategies that suppress relapse while reducing reliance on steroids.

Rituximab is a monoclonal antibody against the B-lymphocyte CD20 antigen and has shown efficacy in the treatment of several autoimmune disorders. Since 2004, there have been some reports showing that administering rituximab to patients with refractory nephrotic syndrome can lead to remission and reduce or eliminate steroid and immunosuppressant drug use^2^,^3^,^4^,^5^,^6^,^7^,^8^,^9^,^10^. Besides, there have also been no reports of severe infusion reactions, including anaphylactic shock, which is a particular concern. While the use of rituximab in nephrological indications is increasing for refractory nephrotic syndrome, few reports have evaluated the medical economics of rituximab for either steroid-dependent nephrotic syndrome (SDNS) or frequently relapsing nephrotic syndrome (FRNS). In particular, clinical evaluation reports are rare^11^,^12^,^13^,^14^,^15^, and there has been great variation in the doses of rituximab used in these studies, with regimens ranging from single doses of 500 mg at 6-month intervals^7^,^16^ to 375 mg/m^2^ body surface area once weekly for four weeks^17^. Consequently, it is difficult to draw any robust conclusions regarding the optimal dosing schedule of rituximab.

To ensure optimal distribution of medical resources for SDNS or FRNS, it is vital that pharmacotherapy is evaluated in terms of medical economics and effectiveness. It is desirable, therefore, to form medical policies based on evidence from reliable cost-effectiveness analysis (CEA).

## Results

### Characteristics of the study population

We included 30 cases (21 males, nine females) with SDNS or FRNS. The average age of patients when rituximab was first given was 29.1 ± 11.4 years and the average time from disease onset to rituximab administration was 13.1 ± 7.9 years (Table 1). For males, the average age when first receiving rituximab was 26.6 ± 9.0 years, and the average period from disease onset to the start of therapy was 14.2 ± 6.7 months; for women, the corresponding values were 34.7 ± 14.1 years and 10.3 ± 9.5 months, respectively. Childhood onset (18 years or younger) accounted for five patients of all cases, but only one of the present cohort was a child (age 16 years) when he first received rituximab. There were no skewed, missing, or censored data.

All 30 patients were receiving prednisolone (mean dose 24.21 ± 13.43 mg/day; dose range 5 to 60 mg/day) when treatment with rituximab was started. Other previously used immunosuppressant drugs included cyclosporine (n = 20), mizoribine (n = 6), mycophenolate mofetil (n = 1), and tacrolimus (n = 1). The price of 500 mg rituximab covered by public insurance was 209,585 yen (2,027 USD) as of September 2014. Based on the treatment regimen used, the cost of administering rituximab was calculated to be 3,493 points per month per case (the prescription cost was a trade-off with other drugs, 338 USD per month).

### Changes in the effectiveness parameters

**Figure 1:**
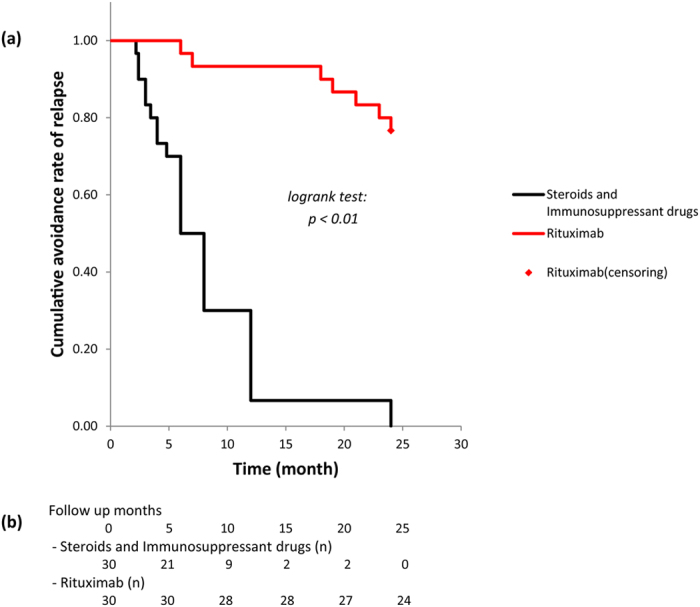
Kaplan–Meier curves of cumulative avoidance rate of the first relapse. (**a**) This estimate was compared before and after administering rituximab, for 24 months in each direction. In this way, rituximab was compared to previous pharmacotherapy. (**b**) Avoidance number of the first relapse.

When analyzing the clinical features before and after rituximab was added, the number of patients with relapse and total number of relapses were significantly lower (30 vs 6; *p* < 0.01, 129 vs 8; *p* < 0.01). Comparison of the total number of relapses between the 24-month period before the first rituximab injection and the 24-month period after the first rituximab injection revealed a significantly lower number of relapses during the latter period (4.30 ± 2.76 times per 24 months vs 0.27 ± 0.52 times per 24 months; *p* < 0.01) (Table 2). When comparing by classification of age group (based on 18 years), there was no significant difference in the relapse rate (4.25 for ≤ 18 years (n = 5) vs. 4.31 for ≥19 years (n = 25); *p* > 0.05). Urinary protein level, which was 2.1 ± 4.6 g/day before rituximab, improved to 0.0 ± 0.0 g/day after rituximab (*p* < 0.05). Although serum creatinine level (0.7 mg/day) did not show a major change from before to after rituximab therapy, serum albumin (3.6 ± 0.9 g/dL to 4.6 ± 0.3 g/dL) and total cholesterol (287.0 ± 112.1 mg/dL to 185.3 ± 38.7 mg/dL) levels did improve (*p* < 0.05). CD20 antigen levels were 7.8% ± 5.2% before rituximab and were significantly reduced to 0.7% ± 1.0% at 24 months after administration (*p* < 0.01). The bone mineral density and T and Z scores were significantly higher at 24 months than at the baseline (0.83 ± 0.15 vs. 0.94 ± 0.13; *p* < 0.05, −1.65 ± 1.38 vs. −0.73 ± 0.78; *p* < 0.01, −1.63 ± 1.41 vs. −0.67 ± 1.01; *p* < 0.01), and the number of patients requiring bisphosphonates was also significantly lower at the end of the 24-month observation period (30 vs 12; *p* < 0.01). The prednisolone dose decreased significantly from 24.1 ± 13.4 mg/day before rituximab to 0.2 ± 0.6 mg/day after administration (*p* < 0.01). Also, the cyclosporine dose decreased from 89.8 ± 64.5 mg/day before rituximab to 12.5 ± 29.1 mg/day after administration (*p* < 0.01). The Kaplan–Meier analysis indicated that patients given rituximab had significantly better prognosis in the cumulative avoidance rate of the first relapse (*p* < 0.01) (Fig. 1).

### Adverse effects

Five patients (17%) experienced adverse effects with rituximab, including three patients who had mild infusion reactions (e.g., cough or hiccough) that did not necessitate treatment withdrawal. One patient developed exanthema almost immediately after starting rituximab therapy, but the pattern was that of a fixed drug eruption on the trunk and it improved following treatment with betamethasone. Another patient developed leukopenia, with their white blood cell count decreasing to 3,000/mm^3^ at 9 months; however, this improved to 5,000/mm^3^ at 12 months. Overall, there were no severe adverse effects during the observation period.

### Change of the cost parameter

**Figure 2:**
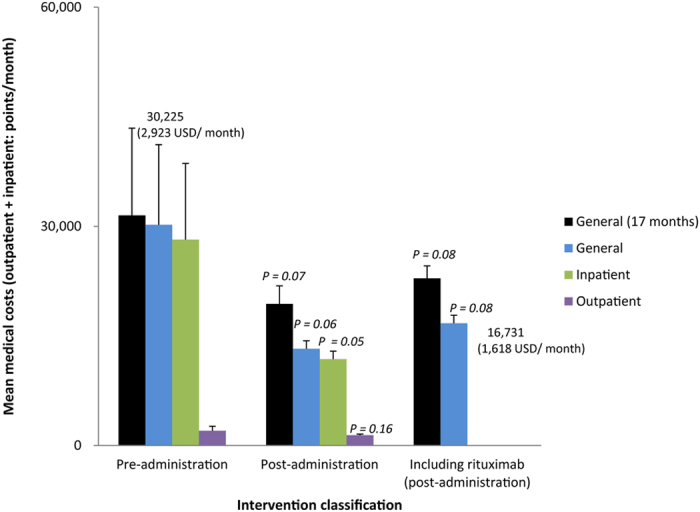
Displacement of medical costs before and after administering rituximab. Even when adding the costs of rituximab, medical costs (at 16,731 general points per month) were much lower than before administering rituximab. Error bars denote standard error. Statistical significance of population mean difference was analyzed using t-test.

The observation period for the cost indicator was 17.8 ± 13.7 months before administration and 29.8 ± 2.6 months after administration (Table 2). When analyzing the average medical invoice fees (total medical costs = inpatient treatment costs + outpatient visit costs) before and after rituximab therapy, the total costs including the costs of the other drugs decreased from 30,225 ± 60,010 points (2,923 USD) per month to 13,238 ± 5,981 points (1,280 USD) per month (general; *p* = 0.06, inpatient; *p* = 0.05, outpatient; *p* = 0.16, Fig. 2). Also, the total medical costs after administration, even when adding the costs for rituximab, were only 16,731 points (1,618 USD) per month, which represented a non-significant decrease in cost from before administration. When the analysis was restricted to ≤17 months (before and after the first rituximab administration), the total cost decreased from 31,493 ± 54,650 points (3,046 USD) per month to 19,397 ± 6,349 points (1,876 USD) per month (*p* = 0.07) (Table 2).

**Figure 3:**
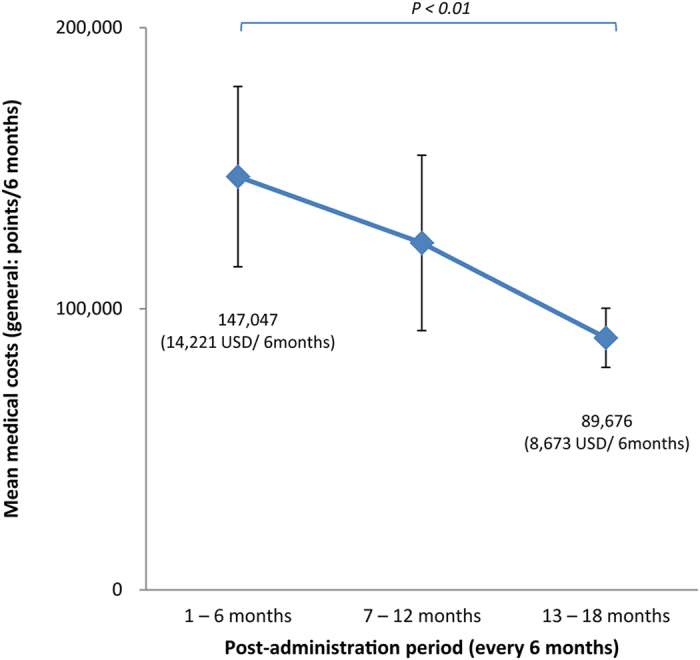
Displacement (6-month cumulative) of mean medical costs (general) after administering rituximab. Medical costs (of adding rituximab) also tended to decrease during the treatment period (comparison of initial period of administration and 1.5 years later; *p* < 0.01). Error bars denote SD. Statistical significance of population mean difference was analyzed using t-test.

**Figure 4:**
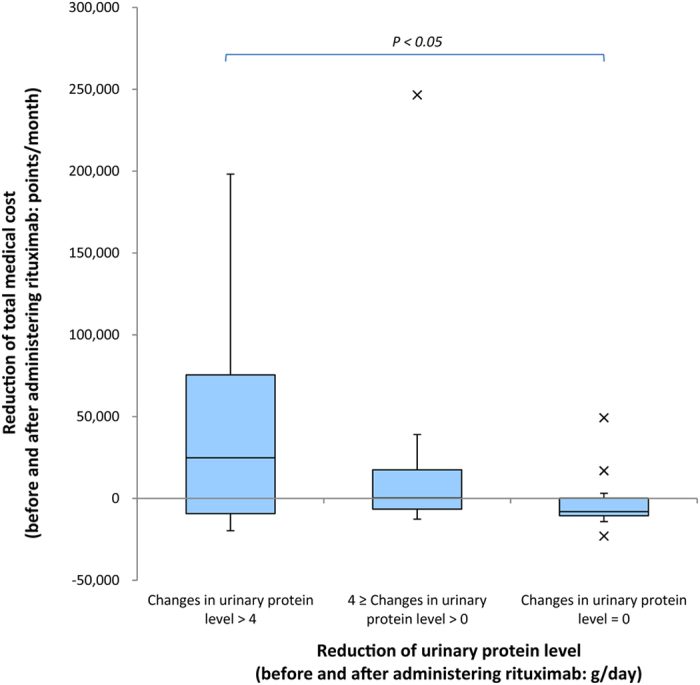
Mutual relationship of urinary protein level and total medical cost (before and after rituximab therapy). Total medical costs decreased with the reduction in urinary protein levels. Notation in the figure is minimum outlier (bottom x) – 5th percentile (bottom whisker) – 1st quartile (bottom of box) – Median – 3rd quartile (top of box) – 95th percentile (top whisker), and maximum outlier (top x). Statistical significance of population mean difference was analyzed using t-test.

The tendency of medical costs to decrease before and after starting rituximab did not show statistical significance for either general case or 17-month case. By contrast, the medical costs after adding rituximab showed a clear trend toward improvement, moving from 147,047 ± 32,054 points per 6 months to 89,676 ± 10,524 points per 6 months: as shown in Fig. 3, this was 14,221 USD per 6 months at the start of the administration period, and 8,673 USD per 6 months after 18 months (*p* < 0.01). The total medical cost also decreased with the reduction in urinary protein levels (changes in urinary protein level >4 vs. changes in urinary protein level = 0; *p* < 0.05, Fig. 4).

### Result of the CEA

Analyzing the cost-effectiveness (the ratio of total medical costs and number of relapses, after correction for the number of months), before and after rituximab therapy, revealed that the cost-effectiveness improved in medical economic terms. This was 317,707 points (30,726 USD) per 24 months (0.27 times) after rituximab therapy compared with 725,403 points (70,155 USD) per 24 months (4.30 times) before therapy (Table 3). Furthermore, this trend was the same after adding the costs for rituximab.

Not only was there evidence of effectiveness, as shown by reduced number of relapses, but there was also evidence of reduced total medical costs, which together showed improved cost-effectiveness. Thus, the pre–post CEA was considered to be dominant (achieving better outcomes at lower cost). For reference purposes, we calculated the total suppression of medical costs over 24 months per single-episode reduction in the relapse rate (Table 3). The reduction amount for total medical costs accumulated over 24 months (including other drugs) per single-episode reduction in the relapse count from before starting rituximab, was 101,082 points (9,776 USD) per 24 months per relapse. Even when adding the costs for rituximab, this figure was 80,297 points (7,766 USD) per 24 months per relapse. In relation to this result, we calculated the pre–post CEA with a ± 50% change from the cost indicator and effectiveness indicator as a sensitivity analysis. Even in the scenario with the lowest cost-effectiveness (when adding the costs for rituximab), it remained in the dominant region with 20,074 points (1,941 USD) per 24 months per relapse; thus, the dominant region had not changed (pre–post CEA > 0). When the analysis was restricted to ≤17 months, even when adding the costs for rituximab, the pre–post CEA was 29,445 points (2,848 USD) per 17 months per relapse (Table 3).

## Discussion

Our aim when performing this study was to clarify, quantitatively by CEA, whether treatment with rituximab for refractory nephrotic syndrome was superior in medical economic terms to previous pharmacological treatments (i.e., administering steroids and immunosuppressant drugs). The results show that rituximab therapy allowed for dosages of steroid and immunosuppressant drugs to be reduced or stopped. When health improved overall, the medical costs of other drugs were commensurately reduced. In particular, we demonstrated that switching from previous pharmacotherapy to rituximab effectively improved the relapse rate. Indeed, when analyzing the clinical features before and after administration, the number of relapses and the levels of urinary protein, serum albumin, and total cholesterol all improved, emphasizing the beneficial contribution of rituximab to the clinical outcome. Concerning the CEA, when we measured effectiveness as the number of relapses, the addition of rituximab produced a dominant CEA (Supplementary Figure S1). Thus, we conclude that cost-effectiveness after administering rituximab was possibly superior to that by conventional approach.

However, this study has some limitations. First, this study is a non-controlled randomized trial, and unfortunately, the small sample in this study meant that we were unable to perform layered analyses based on patient background, such as primary disease, age, or no restrictive protocols for immunosuppressant or steroid dose. Furthermore, the analysis was based on a regimen followed at a single institute, making it possible that the actual state of treatment for nephrotic syndrome in our country was not fully reflected. Moving forward, it will be necessary to perform further large-scale studies in multiple facilities to verify our data. In particular, there is room for further study on the regimen used by adults with nephrotic syndrome. In recent years, when evaluating the medical economic value (i.e., the socioeconomic meaning) of medical technology, some institutions have adapted the CEA to measure patient effectiveness values^18^. Such evaluation aims to organize the effectiveness of medical treatment from a social and economic perspective, which would be an interesting addition to the current knowledge base. Also, the limitations of this study were no inclusion of other costs such as nursing care. With regards to the significance of administering rituximab for SDNS/FRNS, an investigation must be conducted from a wider perspective further; for example, aiming to reduce the burden of nursing care.

Even for economic evaluations of medical technology we should ideally use a randomized controlled design to reduce the influence of potential biases. Pre–post studies have several important disadvantages that have been well described in the literature. These include regression to the mean, time period bias, lack of a control group, and asymmetrical treatment durations^19^. In this study, which was a prospective clinical trial, a scheme was conducted where the cost and effectiveness were compared approximately 24 months before and after switching (adding or reducing the volume of) the administered drug for patients with refractory nephrotic syndrome. For this reason, we cannot exclude that there may have been some influence on the results, however small, by trends in medical technology and medical payment revisions. It is also important that changes in factors such as patient’s age be considered. Although these discussion points were investigated in the sensitivity analysis, it is possible that this may not have been sufficient. Therefore, these issues will need to be covered in future studies. It may also be necessary to validate the results using other designs, such as difference-in-differences design, with higher potential level.

Generally, when the cost-effectiveness is in the dominant region, a pre–post CEA calculation that directly compares two medical technologies (pre-arm and post-arm) is not conducted as with incremental cost-effectiveness ratio^20^. For reference purposes, however, we calculated suppression of total medical costs accumulated over 24 months per single-episode reduction in the relapse count. In another words, we performed verification using a pre–post CEA to determine whether our findings persisted after adding the costs for rituximab. The results showed that even when considering the addition of rituximab-related costs, switching from existing pharmacological agents to rituximab improved medical economic benefits. In addition, the verification by sensitivity analysis was also considered to be sound, giving a pre–post CEA > 0, which remained in the dominant region. Moving forward, it will be desirable to analyze the trends in pharmacological treatment after approval by the medical insurance system and proceed with the verification of the study results.

Rituximab administration for refractory nephrotic syndrome is believed to contribute to preventing its progression through a reduction in the number of relapses and to improved quality of life and reduction in medical costs. The mechanism of this action is unclear; however, there are reports stating that rituximab, by inducing control T cells and control B cells, appears to contribute to immunological tolerance^1^. Furthermore, it has also been reported that rituximab may have the effect of stabilizing the podocyte cytoskeleton and decreasing proteinuria through this mechanism^21^; however, the pathophysiological mechanism underlying the efficacy of rituximab against SDNS/FRNS remains unclear. To perform more exhaustive CEA in this area, it will be desirable to increase this interpretation by elucidating these mechanisms.

In this study, we found that the addition of rituximab for the treatment of SDNS and FRNS significantly improved the clinical outcomes. Moreover, as quantitatively demonstrated by the CEA, treatment with rituximab was possibly superior to previous pharmacological treatments (steroids and immunosuppressant drugs) from a health economics perspective, of which the major determinants were the costs and effectiveness. To confirm these results, we must not only perform additional large-scale trials in multiple facilities but also deepen the interpretation by further elucidating the mechanisms of action of rituximab.

## Methods

### Aims

In this study, we aimed to clarify the health economics of introducing rituximab for the treatment of SDNS and FRNS. Specifically, our intention was to quantify the clinical outcomes and economic impact to determine whether introducing rituximab for patients with SDNS/FRNS was superior to previous pharmacotherapy (steroids and immunosuppressants) in socioeconomic terms. The analysis themes in this study were as follows: (1) analysis of the medical cost fluctuations before and after administering rituximab; and (2) CEA using the number of relapses as the endpoint, and based on the results of a cost analysis.

### Study design and setting

This was a prospective single-arm study performed at a single facility providing treatment for SDNS and FRNS with minimal-change disease at the Department of Medicine, Kidney Center, Tokyo Women’s Medical University. The study was performed from March 2008 to September 2014, and it involved comparison of the same patients for 24 months before, and for 24 months after, treatment with rituximab. This clinical trial was registered with University Hospital Medical Information Network (UMIN) on the 10th of March, 2011, as the title “Uncontrol trial of rituximab treatment on steroid dependent and frequently relapsing minimal cahnge nephrotic syndrome(MCNS)”, registration number UMIN000005231 (interventional, single-arm, non-randomized, open, uncontrolled). We targeted medically insured individuals for analysis from a public insurance perspective. This study was approved by the Institutional Review Board of the relevant medical institution (Tokyo Women’s Medical University: approval number 140201). The patients received explanations based on the Helsinki Declaration before providing their written informed consent, and all methods were performed in accordance with relevant guidelines and regulations. Study perspective was the public medical insurance.

Understanding whether the costs of overall medicine decrease with a change in a particular intervention is often a research question of interest that is assessed using pre–post study. One advantage of pre–post study is that the internal validity of this design is strong (avoiding the need to adjust for selection bias) in naturalistic research^22^. Moreover, it is likely that the sacrifice of external validity is limited because of the drug was administered for patients in the same institution and the disease was characterized by complicating case with the passage of time. Thus, a pre–post study design was used to analyze the initial cost-effectiveness of administering rituximab.

### Study population

We included patients with SDNS or FRNS that appeared during childhood or adulthood and who were receiving at our institution between March 2008 and February 2014. The eligibility criteria for a diagnosis of nephrotic syndrome were urinary protein ≥3.5 g/day (adult) and serum albumin <3.0 g/dL (adult) with edema and lipidosis. Other definitions were as follows: SDNS was defined as the occurrence of relapse during a tapering down period or within two weeks of discontinuing steroid drugs; FRNS was defined as the occurrence of relapse more than twice during a 6-month period. A patient was defined as having a relapse when the trial nephrologists judged it necessary to step up the immunosuppressive therapy, and was supported by a daily urinary protein excretion increasing to ≥3.5 g/day, as judged by a 3+ or 4+ result on a urinary albumin dipstick.

### Outcomes: clinical and economic indicators

#### Primary endpoints

Clinical data after rituximab administration were prospectively recorded, but the clinical data before administration and cost data were retrospectively collected. The clinical data consisted of urinary and serum results, as well as renal tissue images. In this study, the number of relapses was chosen as the main indicator of effectiveness (accumulated during the observation period). Treatment-emergent adverse events, including infusion reactions, are reported by the body system along with the vital signs and pertinent laboratory, chest X-ray, and electrocardiography findings. For the cost data, we used the total amount of medical expenses invoiced in relation to the disease; this included all inpatient and outpatient costs at the medical institute and the amount borne by the patients themselves. In other words, the cost calculation included those costs for initial consultations, repeat consultations, guidance, tests, assessments, imaging tests, image interpretations, prescriptions, dispensing, injections, procedures, treatment, and rehabilitation. For the analysis, material costs for pharmaceuticals and medical devices were organized by their redemption price. Indirect medical costs, such as labor production and transportation costs, were excluded.

#### Secondary endpoint

The secondary endpoint of this study was the CEA, which was based on the pre–post cost-effectiveness analysis (pre–post CEA) calculated as [medical cost (post–pre)/medical effectiveness (post–pre)]^23^. The formula used to calculate is as follows:


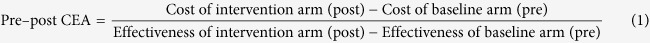


CEA is most useful when we know the outcome of interest and we are determining which of a set of alternative programs or interventions achieves the best outcome for the costs. The pre–post CEA is used to compare the incremental cost and incremental effectiveness in an effort to systematize the economic efficiency of medical interventions. The pre–post CEA also reflects the spread of discussion, by medically and economically measuring the value (meaning) of a medical intervention. When the costs are low and the effectiveness is high for the replacement technology, the pre–post CEA is said to be “dominant”. In this situation, the medical economics of the new therapy are high compared with the comparison therapy, providing evidence that access to the new therapy be promoted.

### Trial protocol

The regimen for this study was 500 mg rituximab every 6 months four times (approximately over 2 years) (Supplementary Figure S2). To minimize the infusion reactions, we administered 4 mg of betamethasone, 20 mg of monoammonium glycyrrhizinate, and 200 mg of acetaminophen to the patients 30 min before the rituximab infusion. Steroid and immunosuppressant therapies at the start of the study were recorded, and an attempt was made to taper the dose or discontinue steroids by 12 months after the first rituximab injection. However, no specific protocol was set for drug tapering, and there was no automatic requirement to discontinue either the immunosuppressant drugs or prednisolone in this trial. Patients were followed up for at least 24 months after the first rituximab injection. The changes in the laboratory parameters, prednisolone dose, frequency of complete remission, frequency of relapse, presence or absence of nephrotic syndrome, degrees of B-cell depletion and repletion, and dosages of prednisolone and cyclosporine were recorded and evaluated at baseline and 1, 3, 6, 9, 12, 18, and 24 months after starting rituximab injections.

As this was a clinical study conducted before medical insurance was applied to patients treated with rituximab, the invoiced medical expenses did not include the costs of rituximab. Therefore, for reference and to investigate whether the results of the analysis changed, we calculated the costs of rituximab after the observation period using the official drug price covered by the insurance service.

### Statistical processing methods

We used *t*-tests for analysis of population mean differences. Moreover, all data are reported as the mean ± standard deviation (SD) unless otherwise noted. The statistical analysis software SAS (Release 9.4, SAS Institute Inc., Cary, NC, USA) was used, and the statistical significance was set to 5%. For some analyses, the 95% confidence intervals were also calculated. Outcome factors for relapse-free time were analyzed by the Kaplan–Meier method as the cumulative avoidance rate for the first relapse, and the results were evaluated with the log-rank test.

In this study, discount processing was not conducted for either the effectiveness or cost indicator. This was because we conducted a short-term evaluation at the midpoint (approximately 24 months after initiating rituximab), and we collected actual data that had a major characteristic of chronic disease. However, two-way sensitivity analyses were conducted (± 50% change) for both the effectiveness indicator and the cost indicator. Costs were analyzed using the number of invoiced medical fee points as the unit. The number of points, which is the unit for the invoiced medical fees, is calculated as one point for every 10 yen. Furthermore, when converting from Japanese yen to US dollars, we used the currency rate as of December 2013 (1 USD = 103.4 yen).

## Additional Information

**How to cite this article:** Takura, T. *et al*. Cost-Effectiveness of Administering Rituximab for Steroid-Dependent Nephrotic Syndrome and Frequently Relapsing Nephrotic Syndrome: A Preliminary Study in Japan. *Sci. Rep.* **7**, 46036; doi: 10.1038/srep46036 (2017).

**Publisher's note:** Springer Nature remains neutral with regard to jurisdictional claims in published maps and institutional affiliations.

## Supplementary Material

Supplementary Information

## Figures and Tables

**Table 1:** Basic characteristics of study targets.

Indicators	Mean ± SD
No. of cases	30
Ratio of males (%)	70.0
Age at start of rituximab therapy (years)	29.1 ± 11.4
Male	26.6 ± 9.0
Female	34.7 ± 14.1
Percentage of those under 18 years of age at the time of rituximab administration (%)	3.3
Time from disease onset to starting rituximab (years)	13.1 ± 7.9
Males	14.2 ± 6.7
Females	10.3 ± 9.5
Childhood (18 years or younger) onset ratio (%)	16.7

**Table 2:** Displacement in clinical characteristics before (baseline) and after (24 months) rituximab therapy.

Indicator	Baseline (mean ± SD)	24 months (mean ± SD)	P-values	Notes
Observation period				
Effectiveness indicator (months)	24.0	24.0	—	Urinary proteins, Creatinine and others
Cost indicator (months)	17.8 ± 13.7	29.8±2.6	—	Cost data was retrospectively collected
Number of relapse (times/24 months)	4.30 ± 2.76	0.27 ± 0.52	<0.01	
18 years or younger	4.25 ± 1.09	0.25 ± 0.43	<0.01	*p* > 0.05
19 years or older	4.31 ± 2.88	0.28 ± 0.53	<0.01	
Urinary protein (g/day)	2.1 ± 4.6	0.0 ± 0.0	<0.05	Baseline was just before rituximab administration, 24 months was after rituximab administration
Creatinine (mg/dL)	0.7 ± 0.2	0.7 ± 0.1	0.709	
	Population mean difference: 0.008, 95% CI; −0.037 to 0.053			
Albumin (g/dL)	3.6 ± 0.9	4.6 ± 0.3	<0.05	
Total cholesterol (mg/dL)	287.0 ± 112.1	185.3 ± 38.7	<0.05	
CD20 (%)	7.8 ± 5.2	0.7 ± 0.1	<0.01	
Bone Mineral Density (g/cm^2^)	0.83 ± 0.15	0.94 ± 0.13	<0.05	
T score	−1.65 ± 1.38	−0.73 ± 0.78	<0.01	
Z score	−1.63 ± 1.41	−0.67 ± 1.01	<0.01	
Prednisolone (mg/day)	24.21 ± 13.43	0.25 ± 0.69	<0.01	
Cyclosporine (mg/day)	89.83 ± 64.5	12.5 ± 29.17	<0.01	
Tacrolimus (mg/day)	0.10 ± 0.54	0.00 ± 0.00	0.223	
Mizoribine (mg/day)	38.33 ± 76.20	0.00 ± 0.00	0.922	
Mycophenolate mofetil (mg/day)	75.00 ± 287.32	33.33 ±126.85	0.704	
Medical fee invoice (general = outpatient + inpatient; points/month)	30,255 ± 60,010 (2,923 USD/month)	13,238 ± 5,981 (1,280 USD/month)	0.064	Including amount covered by patient individual payment
18 years or younger	20,514 ± 22,564	15,151 ± 10,355		
19 years or older	32,167 ± 65,138	12,855 ± 4,944		
(cases in which the analysis was restricted to 17 months)	31,493 ± 54,650 (3,046 USD/month)	19,397 ± 6,349 (1,876 USD/month)	0.067	

Population mean difference was calculated by t-test.

**Table 3:** Medical economics analysis (pre–post CEA) accounting for the medical costs of rituximab.

A. Exclusion of rituximab costs			
Items	Pre-administration	Post-administration	Difference (after–before)
Medical cost difference (points/24 months)	725,403	317,707	−407,696
(USD/24 months)	(70,155)	(30,726)	(−39,429)
Relapse difference (times/24 months)	4.30	0.27	−4.03
Pre–post CEA (points/24 months/times)			101,082
(USD/24 months/times)			(9,776)
Reference: pre–post CEA with a case in which the analysis was restricted to 17 months			
(points/17 months/times)			50,982
(USD/17 months/times)			(4,931)
**B. Addition of costs for rituximab**			
Medical cost difference (points/24 months)	725,403	401,539	−323,864
(USD/24 months)	(70,155)	(38,833)	(−31,321)
Number of relapses (times/24 months)	4.30	0.27	−4.03
Pre–post CEA (points/24 months/times)			80,297
(USD/24 months/times)			(7,766)
Reference: pre–post CEA with a case in which the analysis was restricted to 17 months			29,445
(points/17 months/times)			
(USD/17 months/times)			(2,848)

(Addendum) Analysis corrected for the number of months. Pre–post CEA calculated as [medical cost (post – pre)/medical effectiveness (post – pre)] (Suppression amount for medical costs accumulated over 24 months per one-time reduction (avoid) in relapses, expressed as points per 24 months per time).
