# Supplementary material for: Cost-Effectiveness of Administering Rituximab for Steroid-Dependent Nephrotic Syndrome and Frequently Relapsing Nephrotic Syndrome: A Preliminary Study in Japan
Source: Sci Rep. 2017 Apr 7;7:46036. doi: 10.1038/srep46036 (PMC5384079; doi:10.1038/srep46036)
Supplement: Supplementary Information [file srep46036-s1.pdf]

Supplementary information

The title of this manuscript :

Cost-Effectiveness of Administering Rituximab for Steroid-Dependent Nephrotic Syndrome and Frequently Relapsing Nephrotic Syndrome: A Preliminary Study in Japan

The author list:

Tomoyuki Takura Ph.D., MEng,[1,2] Takashi Takei MD, Ph.D.,[3,4] Kosaku Nitta MD, Ph.D.[4]

Institutions:

[1] Department of Healthcare economics and Health policy, Graduate school of Medicine, The University of Tokyo

[2] Osaka University Graduate School of Medicine

[3] Department of Nephrology, Tokyo Metropolitan Geriatric Hospital and Institute of Gerontology

[4] Department of Medicine, Kidney Center, Tokyo Women’s Medical University

Supplementary Figure S1. **Transition in cost-effectiveness plane (ratio of medical costs and number of relapses) before and after administering rituximab**

Before administration, medical costs were 725,403 points (70,155 USD) per 24 months, with 4.3 relapses. After administration, they were 401,539 points (38,833 USD) per 24 months (with the costs of rituximab), and with only 0.3 relapses. Thus, both the medical costs and number of relapses decreased, demonstrating superior cost-effectiveness after administration compared with that before administration. The analysis was corrected for the number of months.

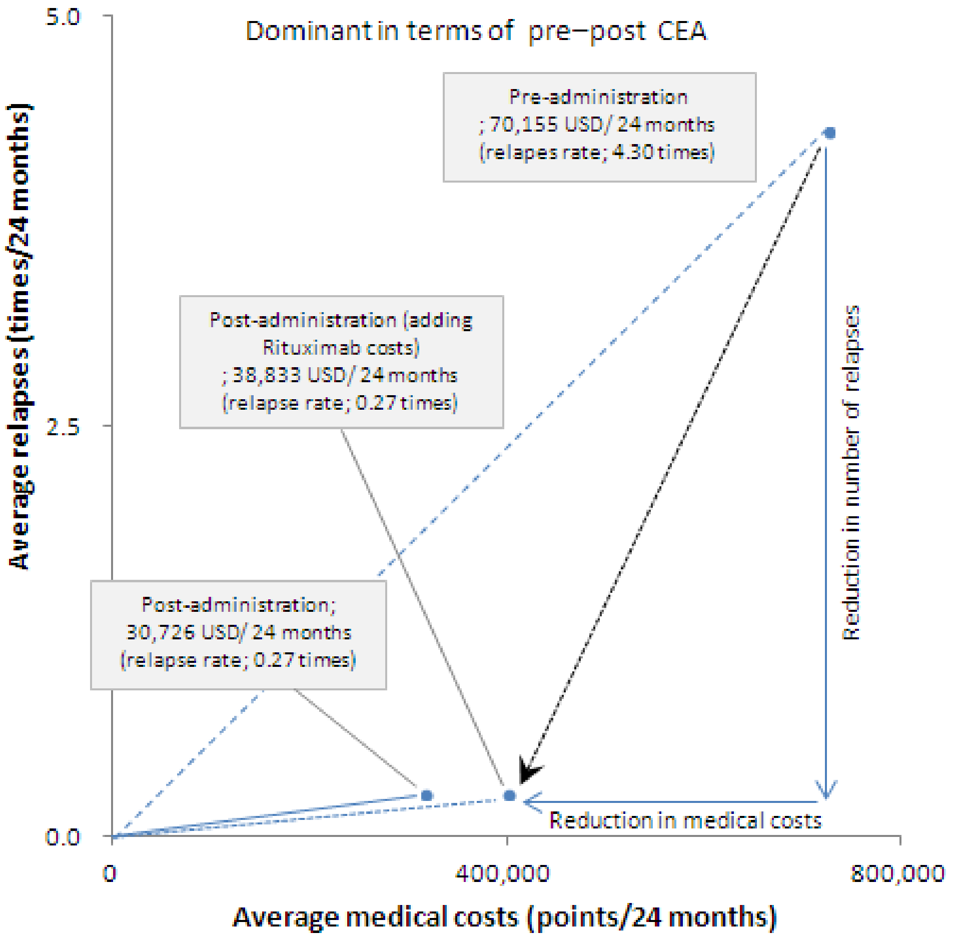

Supplementary Figure S2. **Overview of the regimen used (Image)**

In this study, rituximab was administered four times every 6 months. For the first 6 months from the first dose of rituximab, the dosage of prednisolone and cyclosporine was reduced each month and stopped.

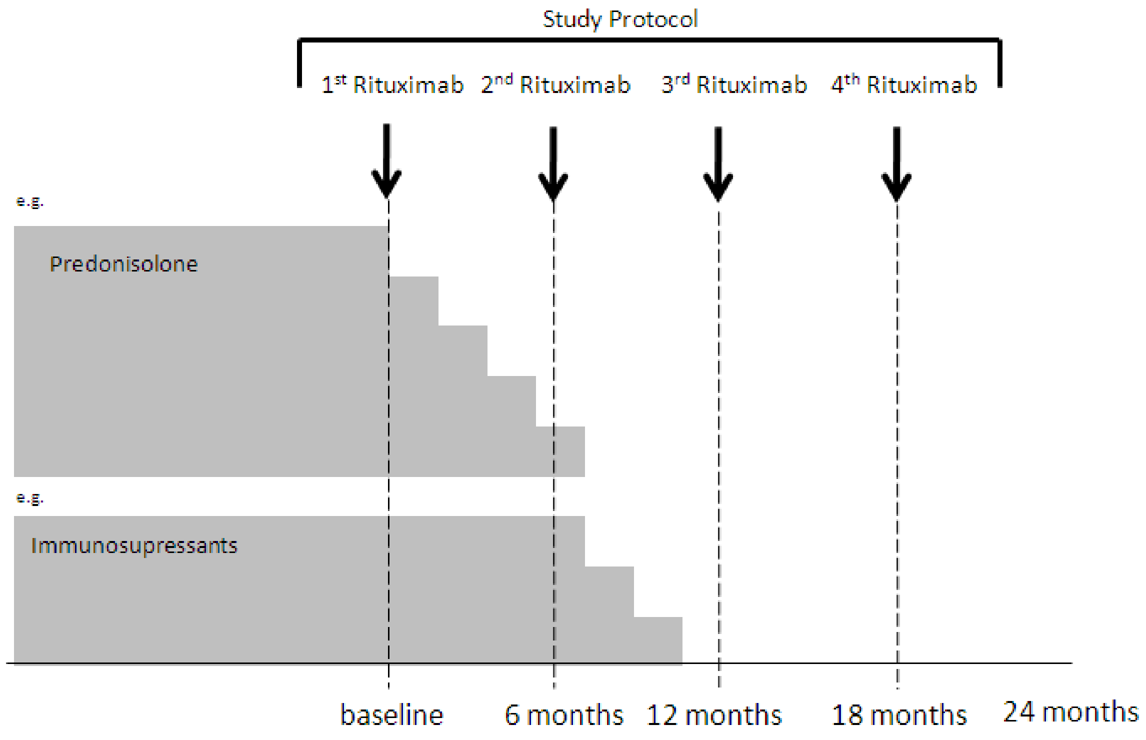

Example of rituximab administration and reduction/stopping of predonisolone/Immunosupressants.
